# Experimental loss of generalist plants reveals alterations in plant-pollinator interactions and a constrained flexibility of foraging

**DOI:** 10.1038/s41598-019-43553-4

**Published:** 2019-05-14

**Authors:** Paolo Biella, Asma Akter, Jeff Ollerton, Sam Tarrant, Štěpán Janeček, Jana Jersáková, Jan Klecka

**Affiliations:** 1 University of South Bohemia, Faculty of Science, Department of Zoology, České Budějovice, Czech Republic; 2 Czech Academy of Sciences, Biology Centre, Institute of Entomology, České Budějovice, Czech Republic; 3 Faculty of Arts, Science and Technology, University of Northampton, Northampton, UK; 4 Department of Ecology, Faculty of Science, Charles University, Viničná 7, Praha, CZ-12844 Czech Republic; 5 University of South Bohemia, Faculty of Science, Department of Ecosystems Biology, České Budějovice, Czech Republic; 6 Present Address: University of Milano-Bicocca, Department of Biotechnology and Biosciences, Milan, Italy

**Keywords:** Community ecology, Conservation biology, Ecological networks, Ecosystem services

## Abstract

Species extinctions undermine ecosystem functioning, with the loss of a small subset of functionally important species having a disproportionate impact. However, little is known about the effects of species loss on plant-pollinator interactions. We addressed this issue in a field experiment by removing the plant species with the highest visitation frequency, then measuring the impact of plant removal on flower visitation, pollinator effectiveness and insect foraging in several sites. Our results show that total visitation decreased exponentially after removing 1–4 most visited plants, suggesting that these plants could benefit co-occurring ones by maintaining high flower visitor abundances. Although we found large variation among plant species, the redistribution of the pollinator guild affected mostly the other plants with high visitor richness. Also, the plant traits mediated the effect of removal on flower visitation; while visitation of plants which had smaller inflorescences and more sugar per flower increased after removal, flower visitors did not switch between flower shapes and visitation decreased mostly in plants visited by many morpho-species of flower visitors. Together, these results suggest that the potential adaptive foraging was constrained by flower traits. Moreover, pollinator effectiveness fluctuated but was not directly linked to changes of flower visitation. In conclusion, it seems that the loss of generalist plants alters plant-pollinator interactions by decreasing pollinator abundance with implications for pollination and insect foraging. Therefore, generalist plants have high conservation value because they sustain the complex pattern of plant-pollinator interactions.

## Introduction

Overall community-level dynamics and ecosystem services are often disproportionately affected by a subset of the local species pool^1,2^. This core of functionally important species contains, among others, generalists, which play a dominant ecological role within the community as they are often among the most abundant species^3,4^ and interact with a majority of other species^5^, mostly by complex facilitation-competition interactions^6^.

Generalist plants offer floral resources, mostly sugars from nectar and proteins from pollens, to a wide spectrum of pollinators and thus help to sustain those pollinators’ populations^7^. A generalist plant is typically visited by both generalist pollinators and specialist ones, which provides multiple advantages. Indeed, visitation by a large number of different pollinators increases the chances of having the pollen dispersed^8^. In turn, when generalist pollinators are foraging in a patch, they can collect resources from a wide range of plants. This strategy could provide nutritional benefits from multiple sources^9,10^, but may also lead to pollination of several plant species because it is believed that generalist pollinators are visiting only a few plant species during each foraging bout^8^. Therefore, conservation of abundant generalists may be important, because their persistence can sustain most of the complexity of interactions taking place in a community^11^.

Despite their functional importance, little is known about the effects of local extinction of generalists. The consequences of species loss have traditionally been investigated by removing species at random, but in nature this is rarely the case as non-random extinctions are the rule^12–14^. In the context of plant-pollinator interactions, the consequences of eliminating species from the community have been studied mostly by simulation models^15,16^. Experimental tests in the field have started only recently and have been so far limited to relatively small manipulations, for instance removing either one invasive plant species^17,18^ or a single native plant species^19^. Other studies have excluded one pollinator species or a small set of the flower visitors^20,21^. Furthermore, only simulation-based studies have tested the effects of excluding several species sequentially^16,22^, while the few field experiments removed only one species and led to disparate results^17,20^. In fact, removing either a pollinator or a plant has different implications. Specifically, by removing a generalist pollinator, more resources will be available for the other pollinators; conversely, the removal of an abundant plant induces an immediate reduction of resources available to the pollinator guild. However, the consequences of the loss of multiple species for plant-pollinator interactions remains an open question.

In this study we experimentally tested the effects of the removal of generalist plant species on plant-pollinator interactions. We focused on pollinators as a guild rather than on individual pollinator species by investigating the effects of the removal of the main floral resources on visitation, nectar consumption, effectiveness of pollination and shifting resource use by the pollinator guild. The experiments comprised two parts. Firstly, a pilot project in the United Kingdom experimentally manipulated a plant-pollinator community by removal of the most generalist plant (from here on called the “pilot study”). This provided a proof-of-concept and suggested ways in which the pollinator assemblage might react to such perturbation. Secondly, in the Czech Republic, we conducted a larger experiment, where we sequentially removed four of the most visited plant species over a short time period to determine how the plant-pollinator interactions would respond to a more profound loss of resources (from here on called the “sequential removal” experiment).

The aim of our experiments was to test whether removal of generalist plants (1) led to a decrease in the overall visitation i.e., the abundance of pollinators in the sites; (2) caused changes of the visitation of individual plant species related to the similarity of floral traits with the removed species, overlap in the flower visitor community, and their level of specialisation, and (3) whether pollination effectiveness (determined by the number of pollen tubes grown in the pistils after visitation) and the amount of used nectar resources (“standing crop”) changed as a consequence of plant removal.

We hypothesised that the pollinator guild could respond to the removal of major floral resources in three ways: (a) they could shift food source and distribute evenly on the remaining plant species; (b) they could shift preferably towards a subset of the plants, possibly on the basis of plant traits; (c) they could stop foraging at the site (or emigrate) due to the lack of the main food source. The scenario (a) might be expected if pollinators are generalists able to use any resources. In addition we hypothesised that under scenario (a) and (b) the plant reproduction, measured as number of pollen tubes, would increase at least in some species, whereas under scenario (c) it would decrease.

## Methods

### Pilot study

The pilot study was conducted in the East Midlands of the UK, in Northampton, during summer 2008 on the 3 ha Quarry Field (52°16′12″ N, 0°52′46″ W), part of the Bradlaugh Fields site, a network of parkland and Local Nature Reserves (data in Table S1).

Surveys of insect visitation were undertaken at two stages: (i) before flower removal in order to determine the plant-pollinator interactions prior to the experiment; (ii) in the days following the removal of inflorescences of the plants species with the highest visitation, i.e. the total abundance of flower visitors on the plant. Inflorescences of the target plant (*Knautia arvensis*) were removed from the whole surface of the entire site. To indicate which plant should be removed, flower visitor abundance was used because it would not be practical to wait for species-level identification of flower visitors needed to decide which was the most generalised plant. We later confirmed that the plant with the highest abundance of flower visitors was also the one with the highest species richness (see Results). During the same period, the nearby Scrub Field, which hosts a vegetation similar to the treated site, was also surveyed two times using the same techniques, as a comparison control site to the Quarry Field. Insect flower visitation surveys were undertaken four times at each of the two stages of the experiment (i.e., before and after removal) between 1 pm and 4 pm on days which were warm and sunny with little or no wind. Surveys lasted 30 minutes and all flower visiting insects seen to be feeding within the flowers were captured along a 2 metre wide belt and within 2 metres in front of the surveyor. The sampling followed a widening spiral from a randomly determined point at a standard pace of 10 m per minute (which makes each survey of an area of approximately 600 m^2^). This method allowed for a large area to be surveyed owing to the relatively low pollinator density where floral resources are patchy and sparsely distributed. Insect specimens were identified either in the field or by experts.

### Sequential removal

The sequential removal experiment was performed in the vicinity of Český Krumlov (South Bohemia, Czech Republic) in the summer 2015. Three experimental sites were chosen (Site 1: 48° 49′ 26.8″ N, 14° 16′ 26.2″ E, area ca. 1500 m^2^; Site 2: 48° 49′ 51.63″ N 14° 17′ 34.12″ E, ca. 1800 m^2^; Site 3: 48° 49′ 35.07″ N 14° 18′ 8.2″ E, ca. 1600 m^2^). The mean pairwise distance between the sites was 2.01 ± 0.95 Km. These experimental sites were small dry meadows surrounded by trees or bushes and with a dense forest on at least two edges. These were intended as physical barriers clearly separating the experimental sites from other habitats in the surroundings. The entire surface of each experimental site was treated by removing the target plant species (see below).

An untreated control site was located at 48° 49′ 26.8″ N, 14° 16′ 26.2″ E, which consisted of habitat and plant community very similar to the treated sites. It was not feasible to pair each experimental site with a control site because of the lack of sufficiently similar sites in the vicinity of the experimental sites, so we opted for a single control accompanying the three experimental sites. An alternative design such as one where we would manipulate one half of a site and use the half as a control would violate the independence of treated or untreated plots given the flying ability of pollinators and the lack of separation of the plots by physical barriers or by distance.

The sampling of insects was based on walking six transects (10 × 1 m) in a randomized order in each site between 9:00 and 17:00 hours. Transects were set up for sampling pollinators and to count plant abundances in order to account for heterogeneity in plant distribution within the sites. The size and number of transects was set proportionally to fit within the small size of the selected sites. All transects were usually sampled twice during each day. Sampling was postponed in the case of rain or strong wind. All insects found while visiting flowers were sampled by a hand net or a mouth aspirator. The floral abundance of plant species was recorded by counting the number of open flowers or compound inflorescences on each plant within each transect. These data were recorded during all stages of the experiment, so that changes in plant phenology across the experimental period would be recorded as changes in the number of flowers, which is the appropriate measure of plant abundance in the context of plant-pollinator interactions. It was not possible to collect further details on the flower sexual maturation stage (pollen presentation or stigma receptivity) because that usually requires destructive methods^23^, which could affect the flowering community in the sites, and it would also not be feasible to collect such data for the entire community given the large number of species present.

In the experimental sites, all flower visitors feeding on flowers were sampled for two days prior to any manipulation (hereafter the “Before” period). At the end of the sampling days, the captured specimens were counted for each plant and thus we were able to determine the plant species with the highest visitation, i.e. the total abundance of flower visitors on the plant. When the experiment was concluded, we sorted the specimens into taxonomical families and morpho-species. This allowed us to confirm that the most highly visited plants in our sites were generalists (Table 1) as the most visited plants in all three sites had averages of 22.15 morpho-species of flower visitors and of 2.35 Shannon diversity index. These values of the richness and diversity of flower visitors are high considering the short-term sampling period and compared to other studies in similar habitats^19,24–26^ and the outcome of the pilot study (see Results). The most visited generalist plant species was then removed from the entire site by cutting all inflorescences in the entire site, as was done in the pilot study. We only removed flowers and left the stems otherwise intact so that the vegetation structure remained unaltered. Twenty four hours after the removal, the sites were sampled again over two days. After we had counted the abundances of the visitors for each plant, we determined the next plant species with the highest visitation, the inflorescences of which were then removed. The flower visitors were sampled again after another twenty four hours for two days. We repeated this procedure until the fourth plant species was removed, which was followed by the last sampling period. Throughout the experiment, we verified that inflorescences of the removed plants were still absent in the sites. Sites 1 and 2 were sampled from 25^th^ June to 12^th^ of July, Site 3 was sampled from 2^nd^ to 17^th^ of July. The control site was sampled synchronously to each of the experimental sites (the same days and during the same hours), but no manipulation of the plant community was performed there (data in Supplementary Information Table S2). For each site, we analysed data on total flower visitation of all plant species by the entire guild of flower visiting insects, data on insect species identity from this experiment are yet not available. The sequence of the plant species removal for both pilot study and sequential removal is detailed in Table 1.

**Table 1:** Plant species removed during the experiments in the experimental sites, with details on the plant family and the pollinators’ abundance (number of specimens) and diversity (number of morpho-species and Shannon Diversity index) found on the given plant.

Treatment period each site	Species to be removed	Plant family	No. of insect specimens	No. of pollinator taxa	Shannon index
**Pilot study**					
Before removing the 1^st^ species	*Knautia arvensis*	Caprifoliaceae	54	11	1.93
**Sequential removal experiment**					
Before removing the 1^st^ species					
In site 1	*Anthriscus sylvestris*	Apiaceae	256	40	2.72
In site 2	*Veronica teucrium*	Plantaginaceae	103	42	3.31
In site 3	*Aegopodium podagraria*	Apiaceae	141	31	3.30
Before removing the 2^nd^ species					
In site 1	*Rubus caesius*	Rosaceae	315	27	1.60
In site 2	*Agrimonia eupatoria*	Rosaceae	96	15	1.04
In site 3	*Veronica teucrium*	Plantaginaceae	26	12	2.37
Before removing the 3^rd^ species					
In site 1	*Knautia arvensis*	Caprifoliaceae	83	22	2.43
In site 2	*Centaurea scabiosa*	Asteraceae	96	34	2.69
In site 3	*Knautia arvensis*	Caprifoliaceae	45	17	2.66
Before removing the 4^th^ species					
In site 1	*Galium mollugo*	Rubiaceae	39	19	2.73
In site 2	*Securigera varia*	Fabaceae	26	4	1.24
In site 3	*Origanum vulgare*	Lamiaceae	55	14	2.48

### Pistil collection and pollen tubes

Pollinator effectiveness was tested by counting pollen tubes in the pistils of flowering plants in two of our experimental sites. Growth of pollen tubes provides information about the effectiveness of the pollination service because it links pollen deposition and seed production^27^, although it may confound cross and self pollination. We collected pistils from on average 40 flowers of each sufficiently abundant flowering plant after each insect sampling period and preserved in formalin-acetic acid-alcohol (FAA) at room temperature. Flowers were collected outside (but nearby) the transects to avoid depletion of flower abundances in the transects. Later, in the laboratory, the flowers were dissected under the microscope and pistils were prepared for softening and staining, following the technique of ^28^. After softening the pistils in 4 M NaOH, they were stained with 0.1% aniline blue in 0.1 M K_2_HPO_4_ for 12 hours. Then, the pistils were washed and mounted in a drop of 50% glycerine on glass slides and covered with cover slips for observation under a fluorescence microscope. Pollen tubes were visible and counted for most of the species. However, in a few cases, pollen tubes were impossible to visualize properly. For these species the number of pollen grains on the stigmas were counted assuming that only germinated grains with tubes still attached would remain on the stigma after the preparation process. All processes were carried out at room temperature. After the observation, the edges of the cover slips were sealed with clear nail polish and stored at 4 °C for future reference. Data were successfully obtained for 10 species because other plant species did not yield any countable pollen tubes (data in Supplementary Information Table S3).

### Measuring the nectar content of flowers and other functional traits

We determined the standing-crop of nectar in flowering plant species at two sites of the sequential removal experiment in order to assess the amount of unused floral resources and its changes after plant removal. To do so, we collected flowers in each site after the pollinator sampling on the same day (data in Supplementary Information Table S4). A 100 µl Hamilton capillary syringe was used for the collection and for washing the nectar into distilled water. Flowers were selected randomly but outside the sampling transects in order to avoid impoverishment of floral resources in the transects; only flowers in full anthesis were sampled. Nectar samples were stored in a cool bag in the field and in a −20 °C freezer in the laboratory. Sugar analysis of nectar was done using high performance anion exchange chromatography with pulsed amperometric detection using a Dionex ICS-3000 system and CarboPac PA1 analytical column. Nectar amount was expressed as milligrams of sugars per flower. Because the method is not sensitive when the amount of sugar is extremely low, several nectar samples from a known number of flowers (of a given species on a given day) were merged in one unique sample which was analysed as described above; afterwards, the amount of sugar per flower was calculated by dividing the quantity of sugar by the number of flowers included in the sample. Data were obtained from an average of 45 flowers per plant species.

Furthermore, we measured several additional functional traits for each plant species in the two sites. The daily production of nectar was measured with a similar methodology as for the standing crop but the only difference was that flowers were bagged for 24 hours before sampling nectar: several inflorescences were bagged in the morning and sampled the next day in the morning, yielding nectar data for on average 45 single flowers per species. This is an appropriate method used for comparing the cumulative secretion of nectar over a standard amount of time which covers the entire daily rhythm in several different species. Other traits were: plant height (linear distance between the ground and the top of an inflorescence, measured on an average of 12 plants per species); inflorescence maximum size (the maximum dimension of the inflorescence, measured on an average of 10 flowers per species); dominant colour of the corolla (categorical variable: “white”, “blue”, “pink”, “yellow”); flower shape coded according to^7^ as follows: bell- or funnel-shaped, dish- or bowl-shaped flowers, flag-shaped flowers, gullet-shaped flowers, head- or brush-shaped, tube-shaped flowers; unclear cases were checked with^29^.

### Statistical analyses

All data were analysed by means of generalized linear mixed-effect models (GLMM) using the library *lme4*^30^ in the R environment^31^ and confidence intervals were estimated with 1000 bootstraps with the function *bootMer*.

#### Site level visitation

For the sequential removal, the overall visitation was derived by calculating the sum of flower visitor abundances across plant species for each transect, separately at each sampling event. Thus, the number of flower-visiting insects per transect was the response variable, treatment was a categorical predictor describing the number of removed plants (“Before”, “1 sp.”, “2 spp.”, “3 spp.”, “4 spp.”). Transect identity nested within site identity was used as a random effect on the intercept and a Poisson distribution was used. We used an offset in the model to account for possible confounding effects caused by factors other than our experimental manipulation, e.g. phenology, decrease of insect abundance due to sampling, etc. To do that, the plant visitation in the control site at a given time (geometric mean across the control site’s transects) was included in the model as an offset. In the experimental sites, a multiple comparison tests were performed using the package *multcomp* with the function *glht*^32^ to compare a given treatment level with the preceding treatment level (e.g. “1 sp. removed” vs “Before”, “2 spp. removed” vs “1 sp. removed”, and so on) in order to test the significance of relative increases or decreases in pollinator abundances.

For the data from the pilot study, the visitation was analysed in a slightly different fashion because flower visitors were collected along a single transect and insect species were taxonomically identified. Thus in a GLMM with Poisson distribution the number of visits by individual flower visitor species at each plant species was used as a response, treatment was a predictor (“before” and “after” removal of *Knautia arvensis*). Species identity was used as a random factor on the intercept. The analysis included visitation in the control site as an offset for the same reasons as for the sequential removal (see above). Specifically, visitation by an insect species on a plant species in the control site was used as an offset for visitation in the same insect-plant combination in the experimental site. For those insect species that were not recorded in the control site, the mean visitation recorded on that plant species across all visiting insect species at a given experimental time was used. Data from the control site were also analysed in a similar way (but without an offset); i.e. we also tested whether plant visitation changed between the sampling periods in the control site. For the control site, changes in the visitation on the species that was removed from the treated site were explored with a similar GLMM model, but without plant species as the random intercept.

#### Plant species level visitation

To test the effect of treatment on each plant species in the sequential removal experiment, plant-level visitation (visiting insect abundance for each plant species at each sampled transect, including unvisited plants) was the response variable and treatment was a predictor variable of the removal events (“Before”, “1 sp.”, “2 spp.”, “3 spp.”, “4 spp.”). An offset of plant abundance measured as the number of flowers per transect was included, to account for possible variation of the amount of flowers during the experiment (changes in plant abundance or phenology), as suggested in^33^. Transect identity within site identity was used as a random intercept. Plant species identity was used as another random factor affecting the slope of the treatment; i.e. we assumed that different plants may respond to the removal treatment in a species-specific way. Poisson distribution was used in the GLMMs.

#### Traits analyses

To test if foraging during the treatment would be directed towards flowers being similar or dissimilar to the removed species, we assessed how visitation related to differences between plant-species’ traits and the removed plants’ traits in the two sites where traits were measured (data in Supplementary Information Table S5). To do that, the log-ratio difference between a given continuous plant trait value and the trait value of the removed plant species, *log(trait*_*sp. i*_/*trait*_*removed sp*._), was calculated for each treatment level and used as a predictor variable. This measure of difference between quantitative values thus varies from negative values, through zero to positive values and has favourable statistical properties for analysis^34^. For categorical variables, a binomial variable was used, i.e. “same” as or “different” from the removed species. We aimed to compare visitation of individual plant species before and after each removal event (i.e. 1 sp. removed vs. before, 2 spp. removed vs. 1 sp. removed, etc.), so we reorganised the data for this analysis with the treatment coded simply as “before” and “after” removal. Plant traits used as predictors of visitation changes were flower colour, flower shape, inflorescence size, plant height and the daily production of sugars in nectar. We tested whether the change in visitation of individual plant species was affected by its traits by including interaction terms between the traits and the treatment variable. By including the treatment as predictor, the variation of visitation amount over the experimental time is included in the model. The number of flowers of each plant species was included as an offset to account for possible variation of plant abundance across treatments. Transect identity within the site identity was used as a random intercept. Plant species identity was used as another random factor affecting the slope of the treatment effect. A Poisson distribution was used in the model. The control site was not included in this analysis because species were not removed in that site and thus it is not possible to calculate the trait similarities between the removed and remaining plant species.

#### Plant-pollinator interaction indices for plants

To test if the redistribution of visitation after removing the generalist plants depended on the interactions of the plant community with the flower visitors, we calculated several plant-pollinator ecological indices for the plants of all three sites, based on the morpho-species data. These indices were: the Pianka’s niche overlap^35^, to measure the overlap in insect fauna between the removed plants and each of the other plants; the normalized degree, that is the number of flower-visitor morpho-species interacting with (collected on) each plant species divided by the total number of flower-visitor morpho-species collected at the site in a given treatment phase; the complementary specialization d′, which is a standardized measure of pollination specialization. These indices were calculated in the R environment with the bipartite^36^ and EcoSimR^37^ packages.

These indices were included as predictors in a GLMM analysis with similar formulation as the one with plant traits. Hence, the visitation on each plant species was the response variable, the treatment simplified as “before” and “after” each plant removal in interaction with the ecological indices of normalized degree, pollinator fauna overlap and complementary specialization were predictor variables. The number of flowers of each plant species was included as an offset to account for possible variation of plant abundance across treatments. Transect identity within the site identity was used as a random intercept. Plant species identity was used as another random factor affecting the slope of the treatment effect. A Poisson distribution was used in the model.

#### Pollination effectiveness

We tested whether the number of pollen tubes in pistils of each plant species, i.e. viable pollen grains deposited and successfully germinating, changed during the experiment in the sequential removal sites. The number of pollen tubes was used as a response variable, the number of removed plants (coded as a categorical variable: “Before”, “1 sp.”, “2 spp.”, “3 spp.”, “4 spp.”) and the plant’s mean visitation across each transect were used as predictors. Random slopes of predictors were included with site identity within plant species as a random factor; i.e. we assumed that the effect of the predictors varied between plant species and sites. The GLMM was fitted with a Poisson distribution.

#### Standing crop of nectar

To test the variation of standing crop of nectar, the amount of sugars per flower of each plant species was used as a response variable, the treatment of the removal events (“Before”, “1 sp.”, “2 spp.”, “3 spp.”, “4 spp.”) and the plants’ mean visitation across transects were used as predictors as in the analysis of pollen tubes (see above). We used site identity within plant species as a random factor affecting the intercept, i.e. assuming that the mean amount of sugar per flower varies between sites and plant species (including the effect of the random factors on the slope was not possible due to a lack of convergence of the model). Gamma distribution was used, and the link function was the natural logarithm.

### Ethics statement

No permits were required for this project in the Czech Republic, because no protected species were affected and the study was conducted on public land. In the UK, JO & ST obtained permission from The Wildlife Trust for Bedfordshire, Cambridgeshire, Northamptonshire and Peterborough to work at Bradlaugh Fields.

## Results

In the pilot study, the surveys identified a total of 13 insect pollinated plant species in flower and 25 pollinating insect species in the experimental site. The plant with the highest pollinator abundance and also the highest visiting species richness was *Knautia arvensis* (details in Table 1). Most other plants were visited by fewer than 20 insect individuals and less than 8 species in both experimental phases, except *Centaurea nigra* which was visited by 41 individuals of 11 species before removal and 86 individuals of 11 species after removal of *Knautia*.

In the sequential removal experiment, the sites 1, 2 and 3 were surveyed for a total of 31, 26 and 23 insect pollinated plant species, respectively. The amount of insect specimens and the sequence of removed plants is detailed in Table 1.

### Results from the pilot study

**Figure 1:**
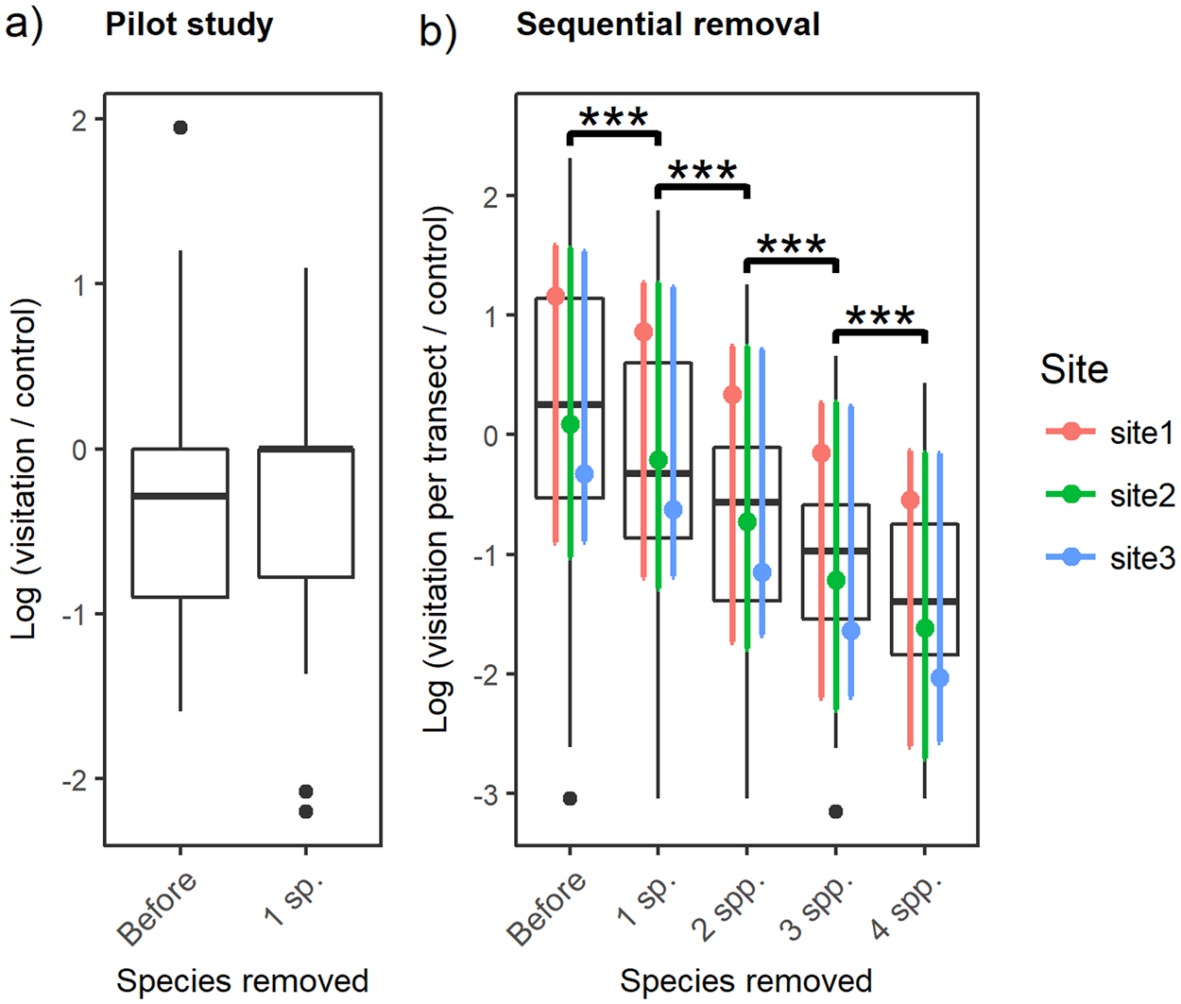
Overall visitation in both pilot study and sequential removal experiment, including offsets of control sites’ visitation. (**a**) Boxplots of raw data of the pilot study indicating the flower-visitor visitation (abundances) for each plant species; (**b**) Plot of the sequential removal experiment where the boxplots indicate visitation across plant species of the same sampling event (i.e. a transect walk) and the estimated (modelled) means and confidence intervals are represented. All plots are on a logarithmic scale. When significant after multiple comparison test, it is indicated with the following codes: ***p < 0.001, **p < 0.01, *p < 0.05.

In the pilot study, visitation to plants changed only slightly after removing one species (Fig. 1). The treatment was not a significant predictor of visitation in the statistical model including an offset of the control’s visitation when compared with a model without treatment variable as reported by a log-likelihood ratio test (**χ**^2^ = 0.194, df = 1, p = 0.66). In detail, the abundance of pollinators on the previously second-ranked generalist species more than doubled in the post-removal phase compared to the pre-removal phase (visitation before = 46, visitation after = 86 flower visitors) while the remaining plants did not gain visitation.

Visitation changed only slightly in the near control site, the difference was not statistically significant (**χ**^2^ = 0.85, df = 1, p = 0.36) (Fig. S1). In the control site, visitation to *Knautia arvensis* (the plant that was removed in the experimental site) did not increase over the study period when compared with a model without treatment variable (**χ**^2^ = 3.60, df = 1, p = 0.06).

### General pattern of visitation in the sequential removal experiment

In the sequential removal sites, visitation per transect decreased sharply after the removal of selected plants. The treatment was a significant and very strong predictor when compared with a model without treatment variable as indicated by a log-likelihood ratio test (**χ**^2^ = 1605.3, df = 4, p < 0.001), in the model including the visitation in the control site as an offset. The model without offset of the control site’s visitation yielded very similar results. Multiple-comparison test of treatment levels gave significant results in most cases (Table 2), with a similar decrease of total visitation after each stage of the removal experiment (Fig. 1).

**Table 2:** Multiple comparison statistics on the effect of treatment on total visitation, i.e. the total abundance of flower-visiting insects per transect, in the sequential removal experiment, with and without an offset with the visitation in the control site.

	Comparison	Estimated trend	Std. Error	P
Without offset of control site’s visitation	1 sp. removed vs Before	−0.071	0.039	0.228
	2 spp. removed vs 1 sp. removed	−0.609	0.049	<**0.001**
	3 spp. removed vs 2 spp. removed	−0.543	0.061	<**0.001**
	4 spp. removed vs 3 spp. removed	−0.195	0.072	<**0.001**
With offset of control site’s visitation	1 sp. removed vs Before	−0.302	0.039	<**0.001**
	2 spp. removed vs 1 sp. removed	−0.519	0.049	<**0.001**
	3 spp. removed vs 2 spp. removed	−0.488	0.061	<**0.001**
	4 spp. removed vs 3 spp. removed	−0.397	0.072	<**0.001**

The treatment of sequentially removing four most visited plant species is coded as “Before”, “1 sp. removed”, “2 spp. removed”, “3 spp. removed”, “4 spp. removed”. Statistically significant effects (P < 0.05) are highlighted in bold.

### Visitation of individual plant species

**Figure 2:**
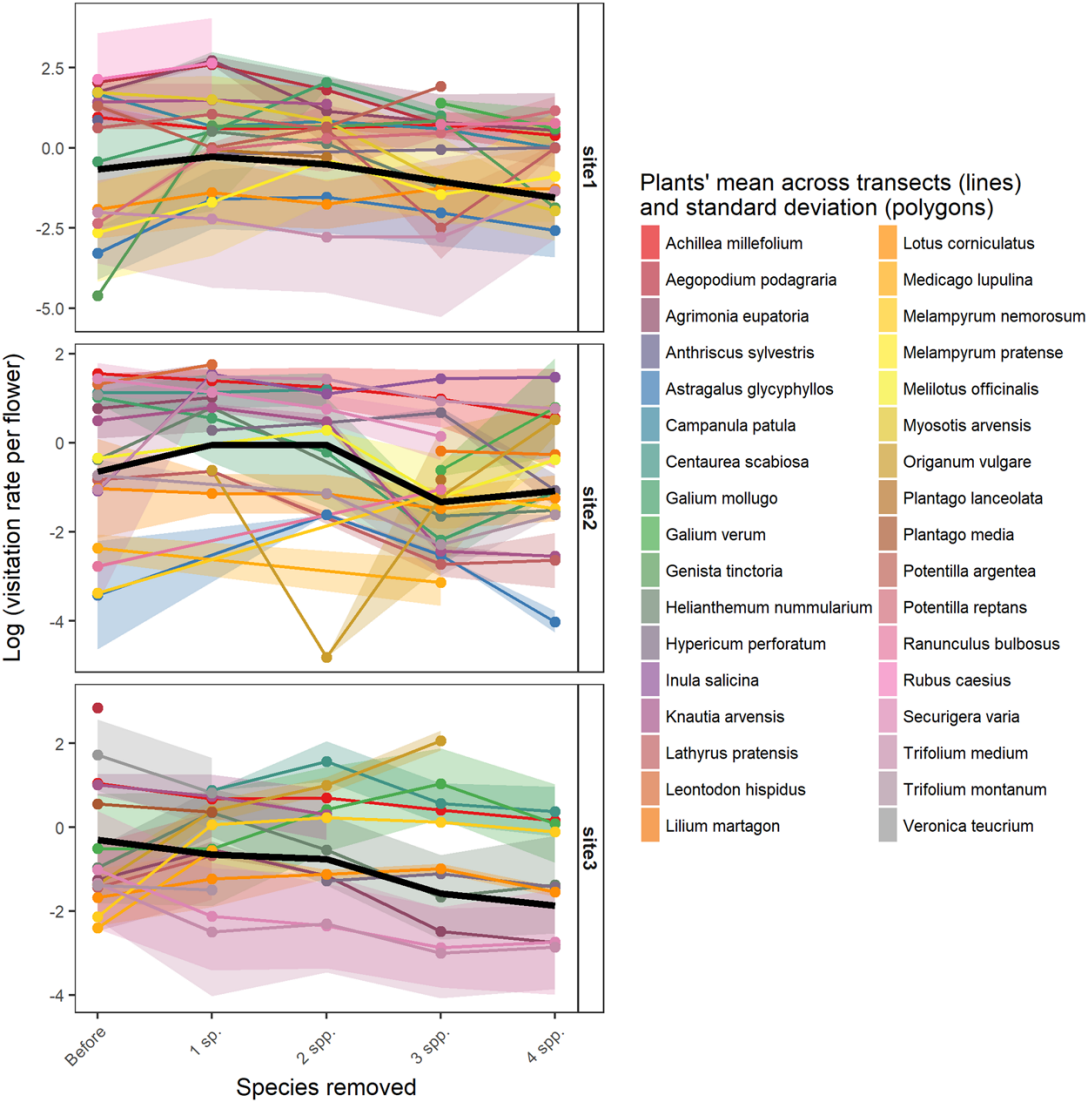
Trends of estimated flower visitor abundances (visitation) per flower of individual plant species in the sequential removal experiment. Visitation is shown on a logarithmic scale, coloured lines are means across transects of a given plant species estimated from the GLMM regression with flower counts per species as offset, coloured polygons are standard deviation around the plants’ estimated means to indicate variation among transects, black line is the plant community average trend.

Visitation at the level of individual plant species was highly variable and significantly dependent on the number of plant species removed during the experiment as reported by a log-likelihood ratio test (χ^2^ = 11.39, df = 4, p < 0.05). The average trend in plant species-level visitation was non- linear across treatment levels and differed between sites. (Fig. 2).

Plants showed high variation in visitation both within sites (among transects) and between sites (coloured lines and confidence intervals in Fig. 2). Nevertheless, the trend was not uniform across plant species, as idiosyncratic responses took place: visitation to some plants increased while in others it decreased in response to the same level of the removal treatment, which could be partly explained by plant traits and morpho-species richness of their visitors.

### Plant traits

**Figure 3:**
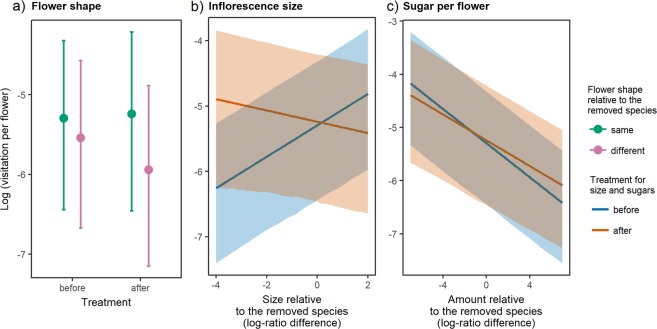
Redistribution of pollinators’ visitation per flower in relation to plant traits relative to the removed species during the sequential removal experiment across all plant removals phases. The visitation per flower including an offset with flower counts (in the GLMM) is represented on the y-axis. On the x-axis, in (**a**) plants with same/different flower shape to the removed species are shown; in (**b**) and (**c**), the log-ratio difference between a given plant trait value and the trait value of the removed plant species is calculated for continuous variables. Means and confidence intervals estimated from GLMM are represented only for the statistically significant traits (plots **a**–**c**).

All plant-traits included in the models except colour and plant height were significant predictors of insect visitation as indicated by the log-likelihood ratio test (Table 3). The redistribution of pollinators was highly dependent on flower size and flower shape but it was less related to sugar content of nectar. Our results show that after the removal of a plant, the visitation remained stable in plants with the same flower shape, but decreased in plants with flower shapes different to the removed species. Furthermore, visitation increased in plants with relatively small inflorescences and high sugar content per flower (Fig. 3, Fig. S2).

**Table 3:** The effect of plant traits on changes of visitation after plant removal evaluated by single-term deletion of interaction terms from a GLMM model.

	ΔAIC	χ^2^	P
Treatment: Color	0.4	2.441	0.628
Treatment: Shape	14.8	16.845	<**0.001**
Treatment: Size	16.5	18.573	<**0.001**
Treatment: Height	−2.0	0.027	0.870
Treatment: Sugars	3.3	5.359	**0.021**

Colons are used for interactions between treatment (“Treatment” referring to before-after removal of one plant species) and difference of trait values of individual plants from the removed species. Trait difference for colour and flower shape was classified as “same” or “different”. Log-ratio difference was used for plant height (“Height”), inflorescence size (“Size”) and amount of sugars in nectar (“Sugars”). ΔAIC refers to the change of AIC after removing the tested term from the full model. Statistically significant effects (P < 0.05) are highlighted in bold.

### Plant-pollinator interaction indices for plants

**Figure 4:**
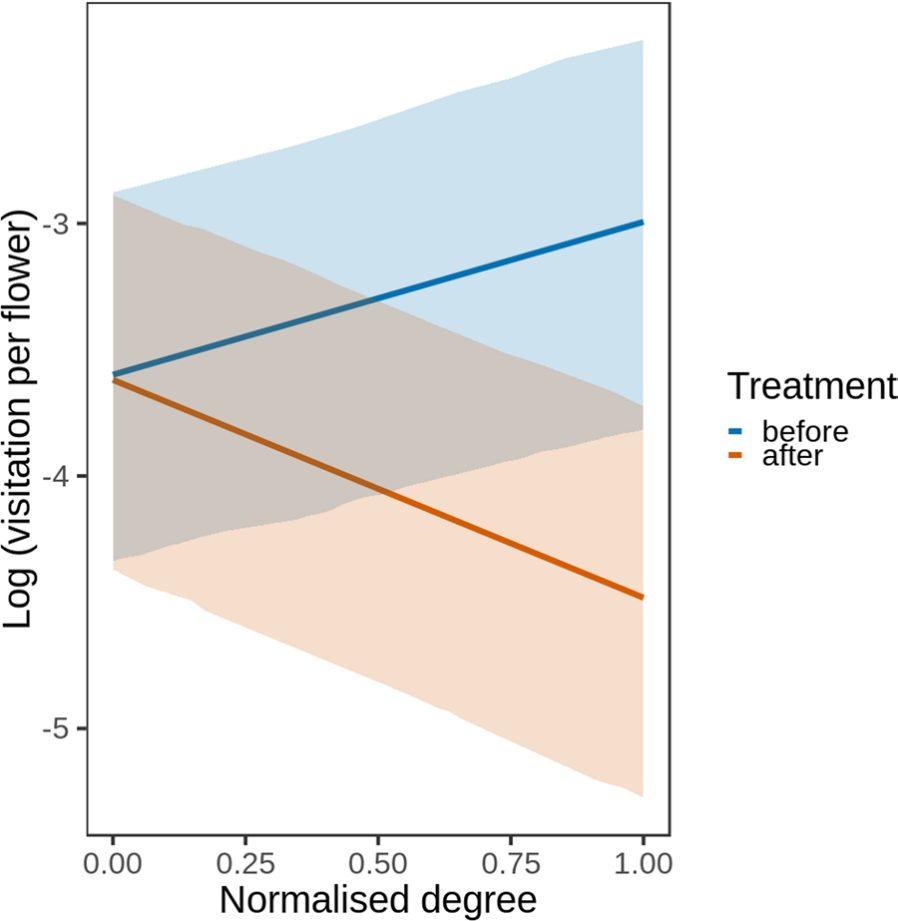
Redistribution of pollinators’ visitation per flower in relation to the normalized degree with flower-visitor morpho-species during the sequential removal experiment across all plant removal phases. The estimated means and confidence intervals of the visitation per flower (including an offset with flower counts in the GLMM) are represented on the y-axis.

Post-removal visitation changes depended mostly on the normalized degree (i.e., the richness of interacting pollinator morpho-species) as indicated by a log-likelihood ratio test (Table 4), while the overlap in pollinator fauna with the removed plants and pollination specialization were not significant predictors and contributed marginally to the visitation changes. Specifically, after removal, visitation decreased more in plants with highest normalized degree (Fig. 4).

**Table 4:** The redistribution of visitation after plant removal and the effect of plant-pollinator interaction indices, evaluated by a single-term deletion of interaction terms from a GLMM model.

	ΔAIC	χ^2^	P
Treatment: overlap	−0.5	1.4162	0.234
Treatment: n. degree	14.9	16.881	<**0.001**
Treatment: d′	−0.1	1.8194	0.1774

Colons represent the statistical interactions between the treatment variable (before-after removal of a plant species) and the indices of flower visitor fauna overlap with a removed plant (Pianka’s niche overlap index), plants’ normalized degree and complementary specialization d′. ΔAIC refers to the change of AIC after removing the tested term from the full model. Statistically significant effects (P < 0.05) are highlighted in bold.

### Pollination effectiveness

**Figure 5:**
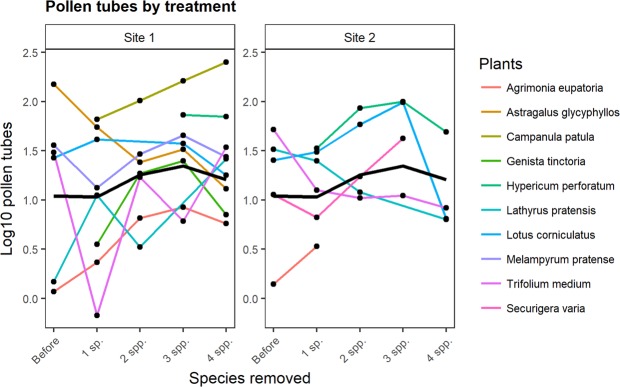
Trends of the number of pollen tubes in two of the sites in the sequential removal experiment. The number of pollen tubes is shown on a logarithmic scale, coloured lines are the estimated means across transects of a given plant species, the black line is the plant community average trend.

The number of pollen tubes per stigma fluctuated significantly during the experiment, but there was no consistent trend (Fig. 5). Log-likelihood ratio test indicated that the treatment was a significant and strong predictor of pollen tube number (**χ**^2^ = 19.9, df = 4, p < 0.001), but visitation was not (**χ**^2^ = 0.62, df = 1, p = 0.43).

The average trend of pollen tube number was not linear but fluctuating (Fig. 5), because it decreased after the first species had been removed but it slightly increased during the following removals, then decreased again after the last species had been removed. However, the trend was not uniform across plant species, as idiosyncratic responses took place (coloured lines in Fig. 5).

### Standing crop of nectar

**Figure 6:**
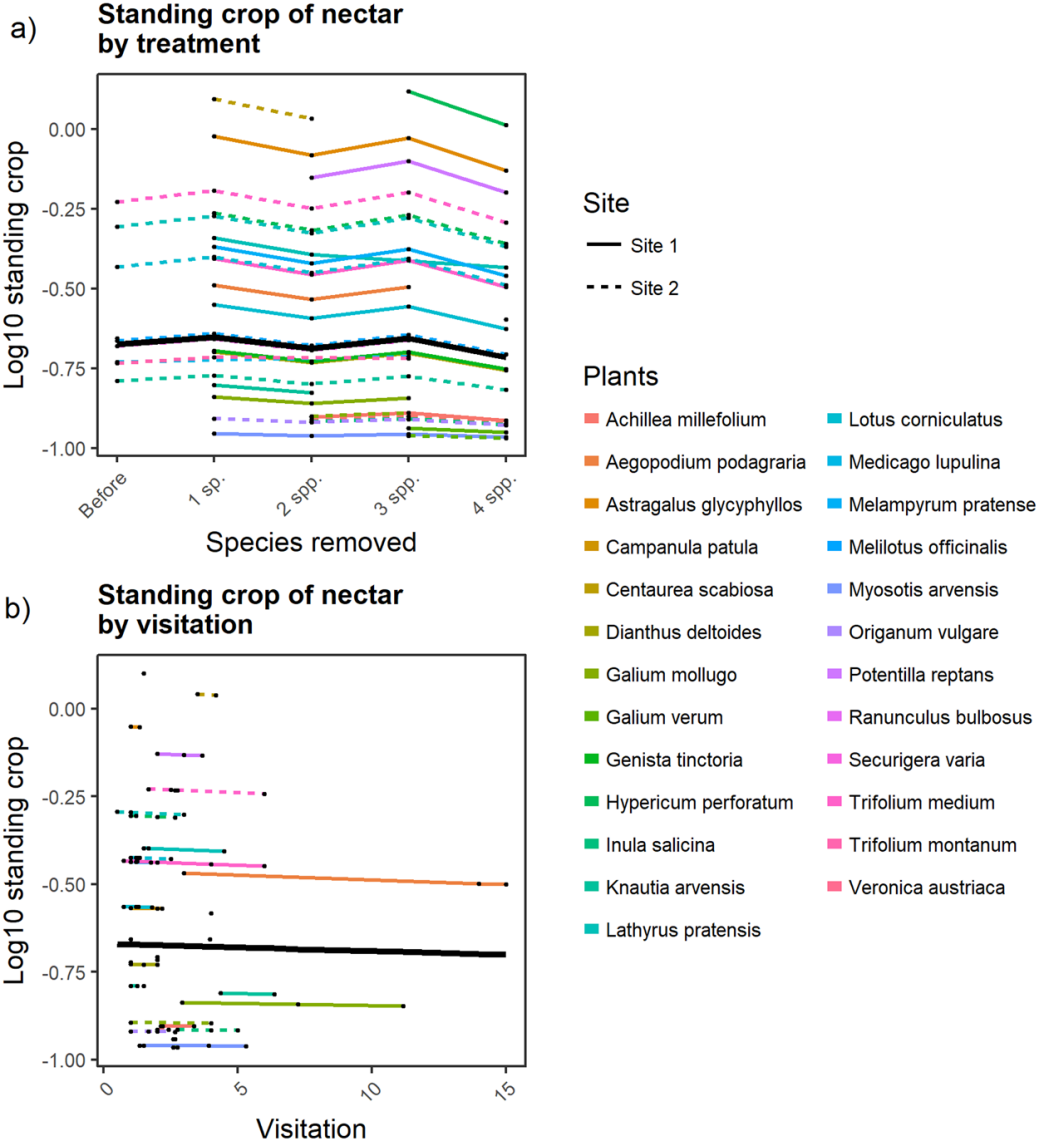
Standing crop in both sites in logarithmic scale vs. Treatment (**a**) and flower visitor’s visitation (**b**), coloured lines are the estimated means across transects of a given plant species, black line is the community-average trend.

The standing crop of nectar did not change significantly as plants were removed (Fig. 6) as reported by a log-likelihood ratio test (**χ**^2^ = 2.14, df = 4, p = 0.71) and visitation had no significant effect (**χ**^2^ = 0.12, df = 1, p = 0.71).

## Discussion

We demonstrated that removal of several generalised plant species led to changes in overall flower visitation at the level of the entire community, but that visitation and pollination of individual plant species were affected in a species-specific way.

We had previously hypothesised that, at the community level, flower visiting insects may respond to the removal of the most visited plants according to one of three scenarios: (a) they may redistribute their visits equally among the remaining plants, (b) they may switch to a subset of the remaining plants depending on the traits of the plants, or (c) they may stop foraging at the affected sites.

Removing one generalist plant species had a limited effect on the pollinators, as in the pilot study, the total visitation did not change after the removal of the most visited plant and actually it was the second most generalist plant species that gained most of the visitation after removal. This shift of pollinating fauna between plant generalists limited insect emigration to neighbouring areas, because the nearby control site changed only slightly and, there, the visitation did not increase on the plant species that was removed in the treated site. Nevertheless, more pronounced changes occurred in the sequential removal experiment, where 4 plant species were removed, one at a time. A decrease of visitation both at the site level (the total visitation per transect) and at the plant assemblage level (as average of visitation across plant species) was recorded after the removal of generalised plants. This suggests that at least some pollinators did not find alternative resources as the removal of plants continued and they stopped foraging at the sites (scenario “c”).

In fact, we have also recorded that changes in visitation depended on how plant functional traits related to the removed plants’ traits. Thus, our data show that flower visiting insects react to the loss of important resources by shifting selectively on a subset of the remaining plants (scenario “b”). Therefore, it seems that a combination of the expected scenarios “b” and “c” actually takes place in response to the loss of key floral resources.

Previous studies theorised that consumers’ adaptive foraging provides stability to the interacting assemblages of species^38,39^. This is because perturbations to the system are buffered by interaction switches that allow using new resources^16,38^. Thus, it is particularly meaningful to investigate how the pollinator guild redistributed after plant removal. In our data, the removal of key floral resources triggered idiosyncratic responses in each plant species in terms of visitation (i.e., plant species with different trends compared to the average community trend, Fig. 2). The redistribution of flower visitors after removal was partly explained by (a) the morpho-species richness of flower visitors interacting with a given plant and (b) the similarity in traits to the removed plants. It seems that plants with a high richness of interacting visitors were the plants that lost most of the visitation; while the plants with low richness of interacting visitors were less affected. This is in contrast with previous studies showing that plants with high pollinator richness would attract even more visitors^24^ or that pollination specialization^19^ or pollinator fauna overlap with the removed plant^40^ could play a role in visitation redistribution after perturbation. Instead, the plant traits apparently constrained the flower visitors in using new resources. In detail, the visitation did not increase in flowers with a morphology different from the removed species (Fig. 3a) and thus a flexible foraging of insects did not emerge in our results, as the visitation seemed constrained by flower morphology. The sugar content of nectar also affected the flower visitors because flower visitors were attracted by more rewarding flowers once the plant community was impoverished (Fig. 3c). Thus, the energetic intake also constrained the process of utilizing new resources. Furthermore, smaller inflorescences also became more visited after removals (Fig. 3b). It is likely that they were underutilised in the original community because of their small floral display, so their visitation benefited from the overall reduction of flower abundance after the removals. Smaller inflorescences may also provide more resources per flower, and thus be more rewarding to flower visitors on a per-visit basis^41^. Taken together, our results imply that foraging can be flexible but also constrained within a specific plant-trait space^42^. These constraints would eventually limit the accessibility of new resources after perturbations.

Lastly, we detected a high spatial variation in pollinator abundances within sites (see the wide standard deviation around plant means in Fig. 2 which reflects differences between transects), that is, a given plant species had high visitation in one sampling transect and low in another transect, even on the same day. Although these patchy responses could be due to a local heterogeneity of abiotic factors^43^, it seems more likely that the plants’ abundances and the set of neighbouring plant species caused variations in responses to generalist removal by shaping competition and facilitation^44–46^. Therefore, it is possible that the spatial structure of the entire plant assemblage could represent an additional factor driving the redistribution of flower visitors after removal. In other words, between a given pair of plant species, competition for pollinators might occur in one patch and facilitation in another patch, which could result in very complex overall patterns^6^.

Another aspect of the complexity that emerged after the experiments is that pollinator service varied across the treatment in a non-linear way, as shown by the fluctuating pattern of pollen tube numbers. This might be due to destabilization of the pollinators by the removal of key resources, which thus reacted in a fluctuating way. In fact, exclusion experiments have highlighted that in the absence of a dominant pollinator, other pollinators can compensate by becoming themselves more effective pollen vectors^21^. However, another study has shown that once the abundant pollinator is excluded, plant fitness and fidelity to flowers can decrease^20^. Although these two studies contradict each other, in fact our results showed both responses: increasing or decreasing pollination effectiveness in different plant species. This resulted from species-specific responses as some plants benefited by receiving more pollen, while other species received less, after removing key plant species. Thus, an idiosyncratic, fluctuating trend of pollination was the outcome at increasing impoverishment of the plant assemblage, as observed also in another study^40^.

The consequences of species loss observed in our experiments have several implications for conservation of biodiversity. Generalist plants which are visited by a wide range of insects play a key role in plant-flower visitor interactions^5,19,47^. The removal of such generalist plants led to important decreases of visitation and significant fluctuations of pollination effectiveness. We also showed that some of the responses were highly variable and species-specific. Furthermore, the insects changed their use of resources after the perturbation but their foraging flexibility was constrained by plant traits, which likely limited the utilization of new resources after plant removal. Taken together, these results suggest that removing generalist plants caused both redistribution to a small portion of the remaining plants (scenario “b”) but also that pollinators decreased overall (scenario “c”). Thus, the stability of this system could depend on a small subset of important species. We thus conclude that generalist plants could benefit co-occurring species and play a key role in sustaining the complex interactions among plant and pollinator assemblages^5,11^. Thus, generalist plants may be more important than commonly thought for the conservation of species-rich ecosystems^48,49^.

## Supplementary information

Supplementary figure S1 and figure S2

Dataset 1

Dataset 2

Dataset 3

Dataset 4

Dataset 5

## Data Availability

All relevant data are within the paper and its Supporting Information files.
