# Supplementary material for: Experimental loss of generalist plants reveals alterations in plant-pollinator interactions and a constrained flexibility of foraging
Source: Sci Rep. 2019 May 14;9:7376. doi: 10.1038/s41598-019-43553-4 (PMC6517441; doi:10.1038/s41598-019-43553-4)

Supplementary Information

Experimental loss of generalist plants reveals alterations in plant-pollinator interactions and a constrained flexibility of foraging

Paolo Biella *[1,2,7] , Asma Akter [1,2] , Jeff Ollerton [3] , Sam Tarrant [3], Štěpán Janeček [4,5], Jana Jersáková [6], Jan Klecka [2]

[1] University of South Bohemia, Faculty of Science, Department of Zoology, České Budějovice, Czech Republic

[2] Czech Academy of Sciences, Biology Centre, Institute of Entomology, České Budějovice, Czech Republic

[3] Faculty of Arts, Science and Technology, University of Northampton, Northampton, UK

[4] Czech Academy of Sciences, Institute of Botany, Třeboň, Czech Republic.

[5] Department of Ecology, Faculty of Science, Charles University in Prague, Praha, Czech Republic

[6] University of South Bohemia, Faculty of Science, Department of Ecosystems Biology, České Budějovice, Czech Republic

[7] Current address: University of Milano-Bicocca, Department of Biotechnology and Biosciences, Zooplantlab, Milan, Italy

*Author for correspondence:
Paolo Biella, email: paolo.biella@unimib.it

Supplementary Dataset includes:

Table S1. Data on the pollinator abundances per plant species from the pilot study

Table S2. Data on the pollinator abundances per plant species from sequential removal experiment

Table S3. Data on the pollen tubes numbers per plant species

Table S4. Data on the standing crop of nectar per plant species

Table S5. Data on the difference between the removed plant species' and the other plants' functional traits

Supplementary figures:

Figure S1. Overall visitation in the experimental sites and in the control sites of the pilot study and of the sequential removal experiment. Boxplots in the left column indicate the visitation (flower-visitor abundance) for individual plant species in the pilot study; The right column shows total visitation (flower-visitor abundance) across plant species for each transect walk in the sequential removal experiment. All plots are on a logarithmic scale.

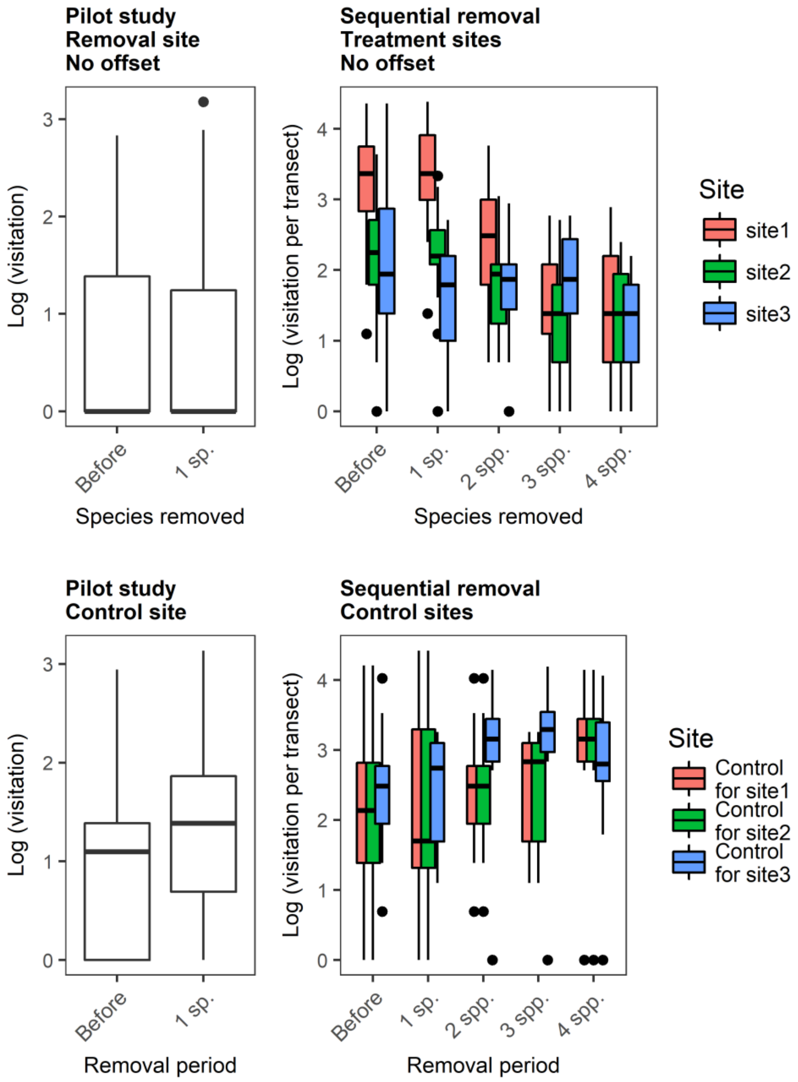

Figure S2. Estimated means for each site and raw data of pollinators' visitation per flower to plant's traits relative to the removed species during the sequential removal experiment including an offset with flower counts in the GLMM. See Fig. 3 for additional information.

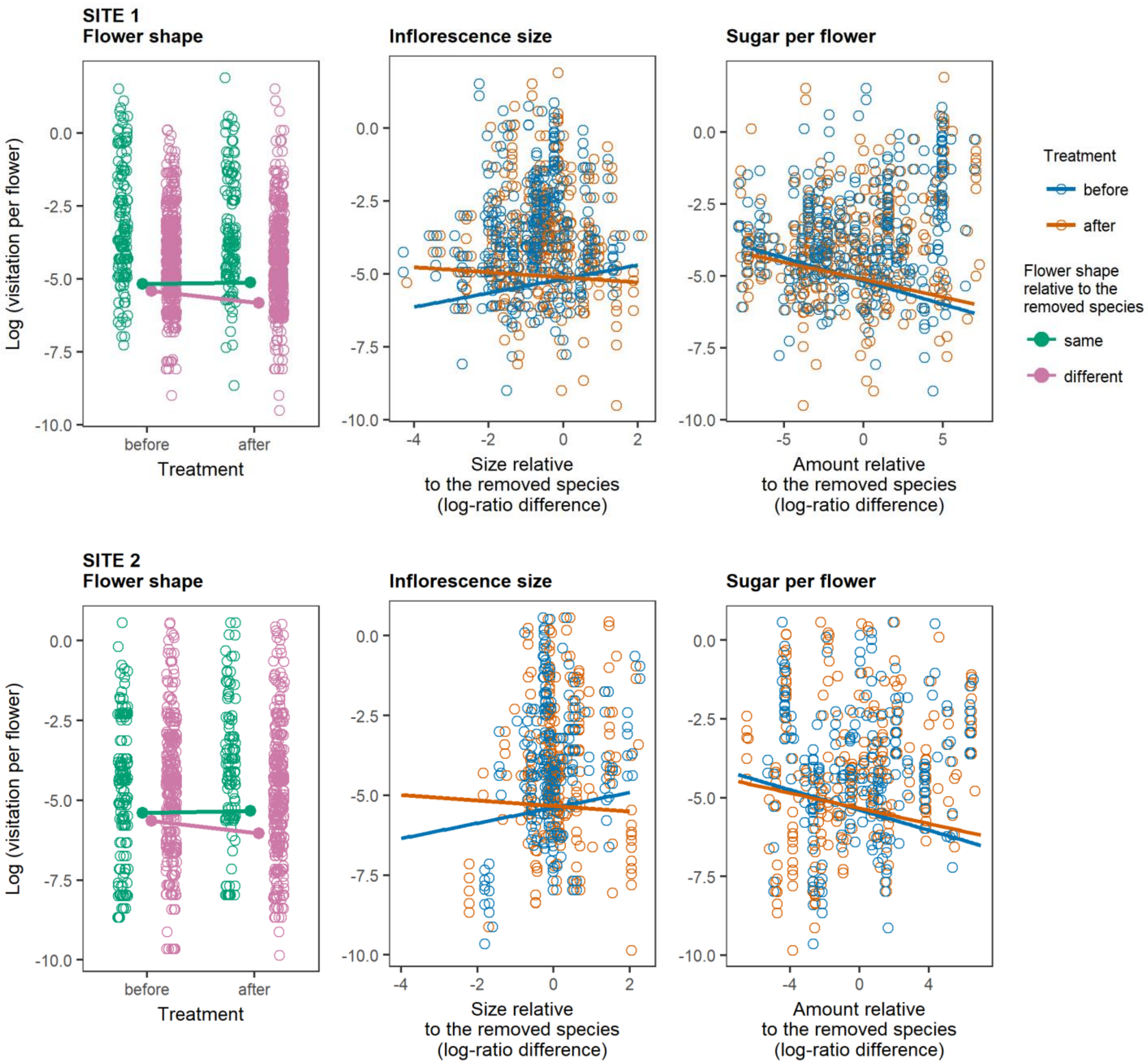

Supplement: Supplementary file 1 — Supplementary figure S1 and figure S2 [file 41598_2019_43553_MOESM1_ESM.pdf]
